# Supplementary material for: Evaluation of antiviral T cell responses and TSCM cells in volunteers enrolled in a phase I HIV-1 subtype C prophylactic vaccine trial in India
Source: PLoS One. 2020 Feb 25;15(2):e0229461. doi: 10.1371/journal.pone.0229461 (PMC7041807; doi:10.1371/journal.pone.0229461)
Supplement: S1 Fig — CD4+ TSCM and CD8+ TSCM cells were gated sequentially on singlets, lymphocyte-sized cells, live CD3+ T cells, CD4+ T cells, CD8+ T cells, and then on memory T cells. Central Memory T cells were defined as CD3+CD4+/CD8+CD45RO+CCR7+, Effector Memory cells as CD3+CD4+/CD8+CD45RO+CCR7-, Terminal Effector cells as CD3+CD4+CD45RO-CCR7- and Naïve T cells as CD3+CD4+/CD8+ CD45RO-CCR7+. TSCM cells were defined as CD3+CD4+/CD8+CD45RO-CCR7+CD28+CD95+. (PPTX) [file pone.0229461.s001.pptx]

## Slide 1
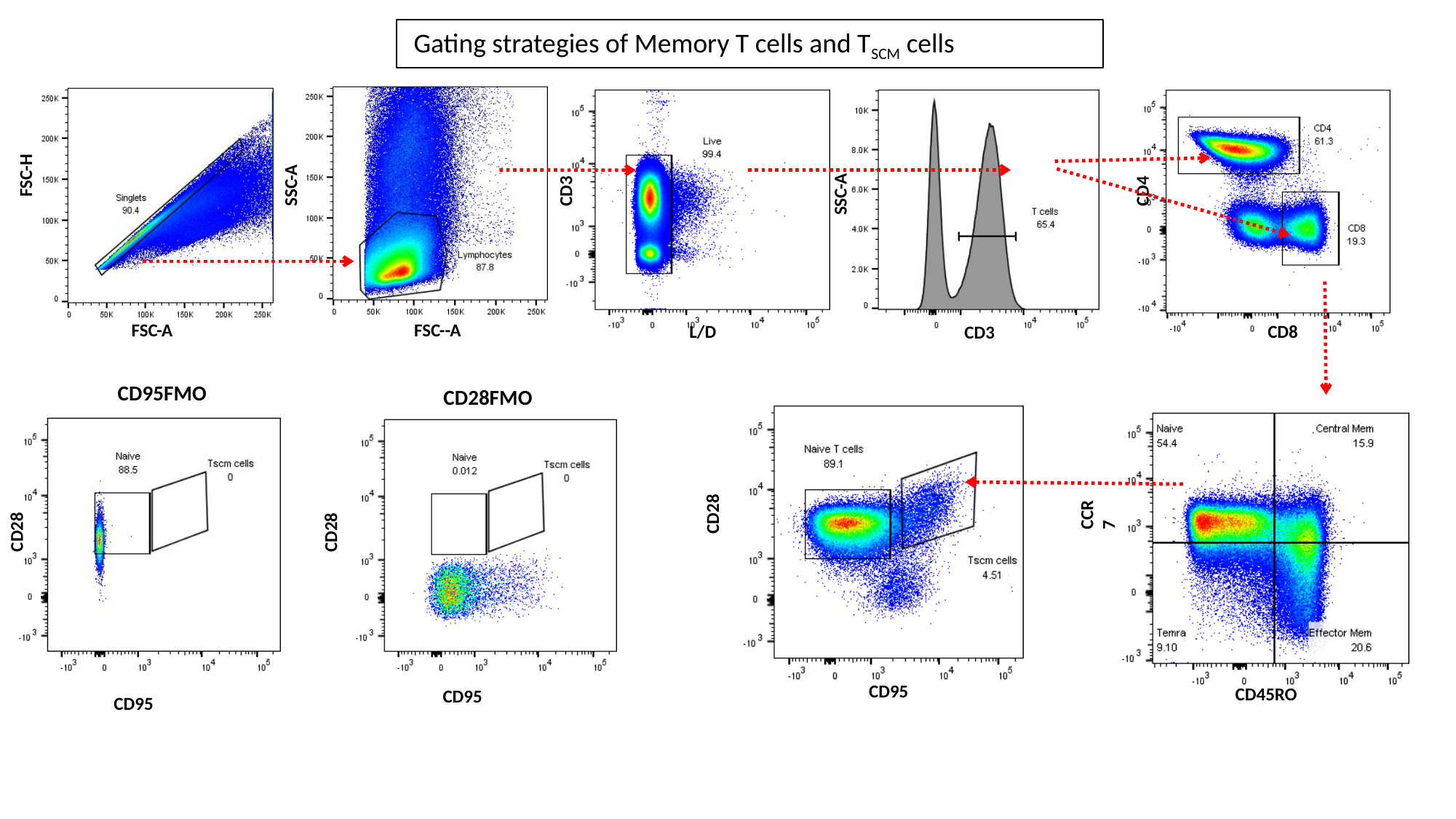

Gating strategies of Memory T cells and TSCM cells
FSC-H
SSC-A
CD3
SSC-A
CD4
FSC-A
FSC--A
L/D
CD8
CD3
CD95FMO
CD28FMO
CD28
CCR7
CD28
CD28
CD95
CD45RO
CD95
CD95
