# Supplementary material for: Evaluation of antiviral T cell responses and TSCM cells in volunteers enrolled in a phase I HIV-1 subtype C prophylactic vaccine trial in India
Source: PLoS One. 2020 Feb 25;15(2):e0229461. doi: 10.1371/journal.pone.0229461 (PMC7041807; doi:10.1371/journal.pone.0229461)
Supplement: S1 Table — (DOCX) [file pone.0229461.s002.docx]

**S1Table.** **Commercial reagents used for multicolor flow cytometry**

Panel for Memory T cells and TSCM Cells:

| **Specificity** | **Flurochrome** | **Ab Clone** | **Vendor** | **Catalog Number** | **Vol/test** |
| --- | --- | --- | --- | --- | --- |
|  |  |  |  |  |  |
| CD3 | APC H7 | SK7 | BD-Pharminogen | 557832 | 2.5ul |
| CD8 | APC R 700 | RPA-T8 | BD-Pharminogen | 565165 | 2.5ul |
| CD4 | PERCP CY5.5 | RPA-T4 | BD-Pharminogen | 560650 | 5ul |
| CCR7 | PeCY7 | G043H7 | BD-Pharminogen | 557648 | 5ul |
| CD45RO | BUV395 | RF8B2 | BD-Pharminogen | 564291 | 5ul |
| CD95 | PE-CF594 | DX2 | BD-Pharminogen | 562395 | 5ul |
| CD45RA | APC | HI100 | BD-Pharminogen | 550855 | 5ul |
| CD28 | FITC | CD28.2 | BD-Pharminogen | 561790 | 20ul |
| CD122 | PE | MIKB2 | BD-Pharminogen | 554525 | 20ul |
| Dead cells | Aqua Blue | -- | Invitrogen | L34957 | 01:20 |
