# Supplementary material for: Evaluation of antiviral T cell responses and TSCM cells in volunteers enrolled in a phase I HIV-1 subtype C prophylactic vaccine trial in India
Source: PLoS One. 2020 Feb 25;15(2):e0229461. doi: 10.1371/journal.pone.0229461 (PMC7041807; doi:10.1371/journal.pone.0229461)
Supplement: S2 Table — (DOCX) [file pone.0229461.s003.docx]

| **S2Table: % Frequencies of memory CD8^+^T cell subsets** | | | | | | |
| --- | --- | --- | --- | --- | --- | --- |
| **Cells** | **Time** | **Placebo (n=4)** | **Group A (n=6)** | **Group B (n=6)** | **Sig.*** | **Sub-group analysis** |
|  |  | **Median (IQR)** | **Median (IQR)** | **Median (IQR)** |  |  |
| CM | Pre-VAC | 4.79 (3.75-5.31) | 7.52 (5.05-9.97) | 5.53 (3.76-8.21) | 0.229 | - |
|  | At the day of VAC -II | 1.36 (1.08-3.43) | 2.76 (1.55-3.37) | 3.08 (1.90-4.33) | 0.475 | - |
|  | 1^st^wk post VAC -II | 4.75 (2.46 8.15) | 4.20 (2.39-5.75) | 2.94 (2.03-5.14) | 0.637 | - |
|  | At the day of VAC-III | 2.96 (2.16-4.40) | 2.68 (2.47-3.31) | 2.19 (1.09-2.61) | 0.423 | - |
|  | 1^st^ wk post VA-III | 3.47 (2.72-4.41) | 2.40 (1.83-3.69) | 2.03 (1.89-2.21) | 0.116 | - |
|  | 2^nd^ wk post VAC-III | 5.27 (3.57-6.24) | 2.73 (2.42-3.66) | 1.39 (1.27-1.70) | 0.013 | B vs. P (0.002); A vs. B (0.028) |
|  | 48^th^wk postVAC-III | 2.33 (1.66-4.39) | 2.37 (2.27-3.52) | 2.62 (1.64 3.52) | 0.927 | - |
| EM | Pre-VAC | 1.92 (1.59-2.67) | 2.71 (2.33-3.47) | 3.74 (2.54- 6.03) | 0.191 | - |
|  | At the day of VAC -II | 1.51 (0.83-1.92) | 7.35 (4.36-7.77) | 4.29 (3.60-8.75) | 0.012 | A vs. P (0.002); B vs. P (0.009) |
|  | 1^st^wk post VAC -II | 2.93 (1.75-4.18) | 7.55 (6.95-8.98) | 8.98 (7.90-9.17) | 0.012 | A vs. P (0.009); B vs. P (0.002) |
|  | At the day of VAC-III | 3.50 (2.79-3.90) | 10.55 (9.87-12.20) | 5.22 (5.14-6.97) | 0.005 | A vs. P (<0.001); B vs. P (0.029) |
|  | 1^st^ wk post VA-III | 2.91 (2.14-3.30) | 11.71 (10.50-12.70) | 7.81 (5.97-9.88) | 0.006 | A vs. P (<0.001); B vs. P (0.019) |
|  | 2^nd^ wk post VAC-III | 2.50 (1.99-2.95) | 13.67 (11.30-19.32) | 8.95 (4.40-12.40) | 0.010 | A vs. P (0.001); B vs. P (0.039) |
|  | 48^th^wk postVAC-III | 2.75 (2.30-3.20) | 15.85 (10.50-16.60) | 13.40 (11.20-15.40) | 0.012 | A vs. P (0.002); B vs. P (0.008) |
| TN | Pre-VAC | 31.50 (25.40-34.60) | 36.95 (28.80-41.70) | 32.65 (25.50-38.32) | 0.479 | - |
|  | At the day of VAC -II | 23.85 (21.40-33.10) | 49.40 (39.70-54.80) | 47.40 (42.60-51.85) | 0.052 | - |
|  | 1^st^wk post VAC -II | 34.55 (21.95-38.70) | 24.45 (18.60-26.72) | 48.45 (35.40-53.50) | 0.025 | A vs. P (0.051); A vs. B (0.003) |
|  | At the day of VAC-III | 28.75 (27.95-33.20) | 38.45 (35.90-44.20) | 48.90 (47.30-51.21) | 0.010 | B vs. P (0.001); A vs. B (0.050) |
|  | 1^st^ wk post VA-III | 23.45 (19.40-27.35) | 37.80 (35.70-39.90) | 40.50 (36.00-49.61) | 0.016 | A vs. P (0.014); B vs. P (0.002) |
|  | 2^nd^ wk post VAC-III | 21.90 (17.10-23.65) | 39.70 (29.30-45.91) | 22.35(20.61-24.30) | 0.004 | A vs. P (0.002); A vs. B (0.002) |
|  | 48^th^wk postVAC-III | 24.03 (16.28-28.85) | 33.15 (31.90-37.51) | 33.11 (25.92-52.10) | 0.151 | - |
| TE | Pre-VAC | 16.45 (14.25-19.15) | 20.70 (14.82-25.70) | 22.11 (15.40-25.81) | 0.288 | - |
|  | At the day of VAC -II | 17.30 (12.95-23.60) | 30.90 (29.41-42.10) | 19.55 (15.30-22.42) | 0.039 | A vs. P (0.010); A vs. B (0.022) |
|  | 1^st^wk post VAC -II | 18.45 (14.45-20.20) | 37.55 (30.55-40.30) | 28.75 (26.10-46.70) | 0.013 | A vs. P (0.002); B vs. P (0.007) |
|  | At the day of VAC-III | 20.10 (14.10-28.45) | 51.10 (42.41-58.80) | 32.62 (23.81-34.83) | 0.004 | A vs. P (<0.001); A vs. B (0.009) |
|  | 1^st^ wk post VA-III | 25.46 (21.35-29.31) | 50.60 (47.60-53.40) | 27.45 (26.01-31.81) | 0.004 | A vs. P (<0.002); A vs. B (0.003) |
|  | 2^nd^ wk post VAC-III | 23.00 (19.45-27.05) | 52.00 (43.50-55.50) | 28.20 (24.30-32.20) | 0.006 | A vs. P (0.001); A vs. B (0.010) |
|  | 48^th^wk postVAC-III | 20.25 (14.40-24.90) | 50.80 (48.90-59.70) | 28.55 (23.30-35.23) | 0.003 | A vs. P (<0.001); A vs. B (0.006) |
| *K-Wallis test was performed to show the difference between Placebo, Group A and Group B. Also, the sub-group by dunn test | | | | | | |
