# Supplementary material for: Evaluation of antiviral T cell responses and TSCM cells in volunteers enrolled in a phase I HIV-1 subtype C prophylactic vaccine trial in India
Source: PLoS One. 2020 Feb 25;15(2):e0229461. doi: 10.1371/journal.pone.0229461 (PMC7041807; doi:10.1371/journal.pone.0229461)
Supplement: S3 Table — (DOCX) [file pone.0229461.s004.docx]

| **S3 Table: % Frequencies of memory CD4^+^T cell subsets** | | | | | | |
| --- | --- | --- | --- | --- | --- | --- |
| **Cells** | **Time** | **Placebo (n=4)** | **Group A (n=6)** | **Group B (n=6)** | **Sig.*** | **Sub-group analysis** |
|  |  | **Median (IQR)** | **Median (IQR)** | **Median (IQR)** |  |  |
| CM | Pre-VAC | 7.86 (6.35-9.26) | 10.73 (9.10-13.20) | 9.91 (9.50-11.90) | 0.083 | - |
|  | At the day of VAC -II | 5.05 (3.95-6.80) | 15.61 (12.8-17.68) | 14.05 (11.70-19.90) | 0.020 | A vs. P (0.006); B vs. P (0.005) |
|  | 1^st^wk post VAC -II | 7.25 (5.08-9.45) | 12.06 (11.10-13.30) | 15.10 (11.30-19.70) | 0.015 | A vs. P (0.020); B vs. P (0.002) |
|  | At the day of VAC-III | 4.08 (2.83-5.85) | 5.18 (3.96-9.18) | 8.52 (7.50-11.20) | 0.056 | - |
|  | 1^st^ wk post VA-III | 5.98 (3.60-8.92) | 7.08 (5.17-11.50) | 15.2 (11.50-17.70) | 0.020 | A vs. B (0.022); B vs. P (0.004) |
|  | 2^nd^ wk post VAC-III | 4.17 (3.35-6.15) | 6.06 (4.95-9.12) | 6.03 (5.00-11.61) | 0.263 | - |
|  | 48^th^wk postVAC-III | 2.97 (2.52-5.75) | 10.62 (6.49-12.10) | 5.10 (4.58-6.63) | 0.528 | - |
| EM | Pre-VAC | 7.35 (3.17-10.00) | 10.85 (1.49-16.61) | 18.50 (10.71-21.00) | 0.075 | - |
|  | At the day of VAC -II | 12.60 (10.85-17.20) | 26.80 (17.40-29.40) | 14.80 (12.20-20.10) | 0.028 | A vs. B (0.019); A vs. P (0.007) |
|  | 1^st^wk post VAC -II | 14.60 (10.75-18.00) | 29.70 (22.80-32.30) | 31.20 (29.20-50.40) | 0.019 | A vs. P (0.022); B vs. P (0.002) |
|  | At the day of VAC-III | 13.65 (11.15-18.10) | 27.12 (13.30-30.40) | 30.65 (27.40-36.60) | 0.120 | - |
|  | 1^st^ wk post VA-III | 14.00 (11.25-15.75) | 33.75 (25.20-38.01) | 29.20 (24.50-37.90) | 0.014 | A vs. P (0.003); B vs. P (0.006) |
|  | 2^nd^ wk post VAC-III | 16.80 (13.45-18.60) | 33.40 (21.70-38.00) | 46.40 (43.90-64.30) | 0.003 | A vs. B (0.012); B vs. P (0.005) |
|  | 48^th^wk postVAC-III | 16.15 (10.01-17.20) | 36.25 (27.50-44.40) | 47.25 (43.10-55.80) | 0.006 | A vs. P (0.019); B vs. P (0.008) |
| TN | Pre-VAC | 52.65 (42.10-54.30) | 54.95 (40.50-65.50) | 47.50 (35.90-51.90) | 0.554 | - |
|  | At the day of VAC -II | 41.25 (42.10-54.30) | 36.70 (32.80-39.90) | 52.00 (32.40-53.50) | 0.353 | - |
|  | 1^st^wk post VAC -II | 50.40 (41.45-57.40) | 29.50 (28.60-34.60) | 29.79 (20.10-47.23) | 0.917 | - |
|  | At the day of VAC-III | 43.20 (35.80-53.30) | 30.25 (23.00-31.60) | 34.55 (29.40-38.80) | 0.057 | - |
|  | 1^st^ wk post VA-III | 48.90 (31.95-63.20) | 30.13 (23.70-35.00) | 44.90 (35.30-48.40) | 0.073 | - |
|  | 2^nd^ wk post VAC-III | 43.65 (30.65-52.00) | 36.80 (25.70-41.30) | 39.74 (36.72-43.80) | 0.609 | - |
|  | 48^th^wk postVAC-III | 37.66 (20.55 50.61) | 48.00 (31.20-66.40) | 44.12 (5.59-52.30) | 0.482 | - |
| TE | Pre-VAC | 25.85 (23.55-29.25) | 29.30 (27.37-36.75) | 31.88 (21.18-36.52) | 0.448 | - |
|  | At the day of VAC -II | 16.22 (14.95-21.51) | 24.55 (23.30-34.21) | 24.31 (9.68-35.49) | 0.448 | - |
|  | 1^st^wk post VAC -II | 16.20 (13.95-18.40) | 35.45 (25.42-36.12) | 33.90 (21.60-34.70) | 0.011 | A vs. P (0.001); B vs. P (0.011) |
|  | At the day of VAC-III | 16.95 (13.80-22.10) | 42.45 (25.80-50.20) | 18.40 (16.04-24.40) | 0.017 | A vs. P (0.006); A vs. B (0.009) |
|  | 1^st^ wk post VA-III | 19.12 (17.10-26.80) | 37.55 (32.50-44.40) | 17.60 (14.20-21.00) | 0.007 | A vs. P (0.022); A vs. B (0.001) |
|  | 2^nd^ wk post VAC-III | 17.10 (13.40-19.65) | 39.70 (32.60-45.80) | 34.55 (22.90-35.40) | 0.013 | A vs. P (0.001); B vs. P (0.032) |
|  | 48^th^wk postVAC-III | 18.81 (15.60-20.65) | 39.31(29.90-45.10) | 38.10 (34.01-40.50) | 0.036 | A vs. P (0.017); B vs. P (0.007) |
| *K-Wallis test was performed to show the difference between Placebo, Group A and Group B. Also, the sub-group by dunn test | | | | | | |
