# Supplementary material for: Evaluation of antiviral T cell responses and TSCM cells in volunteers enrolled in a phase I HIV-1 subtype C prophylactic vaccine trial in India
Source: PLoS One. 2020 Feb 25;15(2):e0229461. doi: 10.1371/journal.pone.0229461 (PMC7041807; doi:10.1371/journal.pone.0229461)
Supplement: S4 Table — (DOCX) [file pone.0229461.s005.docx]

| **S4Table: Total numbers of memory CD8^+^T cell subsets** | | | | | | |
| --- | --- | --- | --- | --- | --- | --- |
| **Cells** | **Time** | **Placebo (n=4)** | **Group A (n=6)** | **Group B (n=6)** | **Sig.*** | **Sub-group analysis** |
|  |  | **Median (IQR)** | **Median (IQR)** | **Median (IQR)** |  |  |
| CM | Pre-VAC | 15011(14069 -17116) | 6882 (5519 -10644) | 12717 (8693 -19133) | 0.075 | - |
|  | At the day of VAC -II | 16501 (10373-25403) | 5302 (2396-7849) | 21904 (20813-22854) | 0.007 | A vs. B (0.030) |
|  | 1^st^wk post VAC -II | 4962 (1808-15663) | 5285 (4991-9958) | 1503 (1256-1810) | 0.067 | A vs. B (0.037) |
|  | At the day of VAC-III | 23930 (17659-40546) | 19339 (15664-27889) | 6915 (5778-7300) | 0.030 | B vs. P (0.027); A vs. B (0.050) |
|  | 1^st^ wk post VA-III | 12337 (8074- 24476) | 17816 (7326-18509) | 8760 (6012-10741) | 0.398 | - |
|  | 2^nd^ wk post VAC-III | 25126 (15944-39344) | 18194 (14562-23481) | 18316 (15993-25209) | 0.526 | - |
|  | 48^th^wk postVAC-III | 27633(19737- 48794) | 22347 (19517-25740) | 27465 (20871-41492) | 0.490 | - |
| EM | Pre-VAC | 15968 (13559-23607) | 19862 (14651- 24079) | 25355 (19598-30894) | 0.407 | - |
|  | At the day of VAC -II | 23001 (18087-32089) | 12728 (5525-16158) | 15298 (13828-22034) | 0.070 | A vs. P (0.030) |
|  | 1^st^wk post VAC -II | 18815 (7395-29468) | 29203 (17676- 41366) | 6739 (5380-8472) | 0.080 | A vs. B (0.037) |
|  | At the day of VAC-III | 12994 (10123- 18931) | 11219 (9733-23424) | 4238 (2722-5688) | 0.073 | - |
|  | 1^st^ wk post VA-III | 8702 (4245-15390) | 9041 (3922-10603) | 6381 (5356-10461) | 0.934 | - |
|  | 2^nd^ wk post VAC-III | 25758 (22808- 28513) | 32453 ( 14427- 43143) | 28296 (19090- 34605) | 0.863 | - |
|  | 48^th^wk postVAC-III | 25481(16016-34946) | 30633(29739-32365) | 11607 (10997-13145) | 0.009 | A vs. P (0.041); A vs. B (0.005) |
| TN | Pre-VAC | 83935 (67484-94110) | 76234 (41033-94286) | 92869 (72908-143642) | 0.612 | - |
|  | At the day of VAC -II | 72116 (64884- 88766) | 75234 (70829- 82049) | 108630 (99695-120362) | 0.299 | - |
|  | 1^st^wk post VAC -II | 50016 (55475- 96616) | 83376 (73106- 101563) | 14282 (12512-18013) | 0.027 | A vs. B (0.011) |
|  | At the day of VAC-III | 82097 (49534- 104093) | 56171( 43517-101384) | 58042 (50157-84467) | 0.836 | - |
|  | 1^st^ wk post VA-III | 52540 (27655- 64515) | 76315 (37522- 86730) | 62106 (48509-73323) | 0.334 | - |
|  | 2^nd^ wk post VAC-III | 87942 (57147- 98182) | 73894 (67495-74903) | 79026 (67548- 83325) | 0.558 | - |
|  | 48^th^wk postVAC-III | 88659 (63398-105373) | 71867 (70736-90905) | 52993 (42939-88838) | 0.041 | - |
| TE | Pre-VAC | 16728 (15202-25406) | 42523 (15874-66224) | 38376 (20619- 41818) | 0.339 | - |
|  | At the day of VAC -II | 57987 (34558 -71912) | 30337 (28970-41072) | 23846 (12212-29476) | 0.109 | - |
|  | 1^st^wk post VAC -II | 43817 (25370-74386) | 39918 (30005-51299) | 16635 (9723-41241) | 0.369 | - |
|  | At the day of VAC-III | 13892 (8436- 22158) | 7162 (5911-8513) | 5241 (4591-7466) | 0.211 | - |
|  | 1^st^ wk post VA-III | 9088 (2809- 25600) | 3928 (3255-5619) | 7602 (3005-8243) | 0.710 | - |
|  | 2^nd^ wk post VAC-III | 18323 (17874-42153) | 6039 (3967-9684) | 32000 (19000- 42015) | 0.004 | A vs. P (0.027);A vs. B (0.003) |
|  | 48^th^wk postVAC-III | 27087 (16131- 36338) | 13408 (5891-14092) | 31871 (28881- 32399) | 0.014 | A vs. B (0.006) |
| *K-Wallis test was performed to show the difference between Placebo, Group A and Group B. Also, the sub-group by dunn test | | | | | | |
