# Supplementary material for: Evaluation of antiviral T cell responses and TSCM cells in volunteers enrolled in a phase I HIV-1 subtype C prophylactic vaccine trial in India
Source: PLoS One. 2020 Feb 25;15(2):e0229461. doi: 10.1371/journal.pone.0229461 (PMC7041807; doi:10.1371/journal.pone.0229461)
Supplement: S5 Table — (DOCX) [file pone.0229461.s006.docx]

| **S5Table: Total numbers of memory CD4^+^T cell subsets** | | | | | | |
| --- | --- | --- | --- | --- | --- | --- |
| **Cells** | **Time** | **Placebo (n=4)** | **Group A (n=6)** | **Group B (n=6)** | **Sig.*** | **Sub-group analysis** |
|  |  | **Median (IQR)** | **Median (IQR)** | **Median (IQR)** |  |  |
| CM | Pre-VAC | 33077 (27914-43465) | 45090 (32690-53139) | 36976 (27224-69082) | 0.816 | - |
|  | At the day of VAC -II | 38619 (30449-46771) | 37605 (29458-39787) | 66429 (61476-74711) | 0.033 | A vs. B (0.021) |
|  | 1^st^wk post VAC -II | 46250 (5528-58335) | 53427 (38843-60990) | 34372 (23166-40333) | 0.229 | - |
|  | At the day of VAC-III | 54867 (36530-70277) | 42512 (18703-58932) | 57896 (37835-71034) | 0.535 | - |
|  | 1^st^ wk post VA-III | 36714 (19090-53569) | 52323 (42258-56619) | 66560 (62361- 95744) | 0.045 | B vs. P (0.029) |
|  | 2^nd^ wk post VAC-III | 44431 (31718- 59798) | 50559 (37429-68324) | 76490 (68536-106805) | 0.016 | B vs. P (0.016); A vs. B (0.031) |
|  | 48^th^wk postVAC-III | 47747 (37321-59635) | 59085 (43905-81144) | 63449 (54870-109010) | 0.761 | - |
| EM | Pre-VAC | 59984 (50787-66130) | 68910 (64560-88981) | 93311 (79341-13567) | 0.066 | B vs. P (0.010) |
|  | At the day of VAC -II | 79510 (55821-128281) | 88989 (68965-102357) | 138580 (83888-159188) | 0.312 | - |
|  | 1^st^wk post VAC -II | 48922 (37140-52798) | 60705 (58616-67598) | 85064 (61907-124772) | 0.021 | B vs. P (0.010) |
|  | At the day of VAC-III | 63148.(43768-76502) | 37935 (22716-44949) | 52318 (43959-55868) | 0.180 | - |
|  | 1^st^ wk post VA-III | 31003 (17617-47277) | 52203 (36329-69348) | 48501 (40177-59499) | 0.182 | - |
|  | 2^nd^ wk post VAC-III | 61838 (50992-72160) | 56419 (28711-69525) | 101391 (77652-116607) | 0.016 | A vs. B (0.009) |
|  | 48^th^wk postVAC-III | 75044 (73522-78460) | 74747 (59404-81080) | 72431 (55841- 98166) | 0.863 | - |
| TN | Pre-VAC | 78260 (63260-93077) | 73155 (36416-93298) | 59159 (32894-104938) | 0.479 | - |
|  | At the day of VAC -II | 64622 (52034-67320) | 89285 (78305-109523) | 77620 (47799-138370) | 0.052 | - |
|  | 1^st^wk post VAC -II | 49670 (12845-88122) | 110804 (101008-139873) | 24062 (19361-30311) | 0.025 |  |
|  | At the day of VAC-III | 68356 (40381- 96013) | 18260 (69789-168573) | 93270 (69604-125703) | 0.010 |  |
|  | 1^st^ wk post VA-III | 73644 (35776- 97719) | 107674 (74276-139178) | 95768 (46335- 167691) | 0.016 |  |
|  | 2^nd^ wk post VAC-III | 77381 (58107-100713) | 128260 (117346-140384) | 102898 (71410-115567) | 0.004 |  |
|  | 48^th^wk postVAC-III | 85722 (65036-107184) | 85700 (77438-104680) | 70792 (46383-102322 | 0.151 | - |
| TE | Pre-VAC | 50015 (48187- 53503) | 52126 (37128- 64595) | 66724 (44554-89123) | 0.652 | - |
|  | At the day of VAC -II | 62940 (52812-110171) | 63176 (45303-71763) | 59704(32659- 117583) | 0.963 | - |
|  | 1^st^wk post VAC -II | 33026 (30499-57140) | 53183 (45789- 67844) | 56519 (44187-79827) | 0.281 | - |
|  | At the day of VAC-III | 17743 (11196 -23283) | 22593 (18802- 29541) | 13095 (5932- 16924) | 0.068 | A vs. B (0.031) |
|  | 1^st^ wk post VA-III | 10267 (5743-14440) | 19009 (18802- 32715) | 8688 (5029-15562) | 0.044 | A vs. P (0.059); A vs. B (0.043) |
|  | 2^nd^ wk post VAC-III | 21592 (15500- 28350) | 27824 (17705- 37742) | 25038 (19070- 26872) | 0.818 | - |
|  | 48^th^wk postVAC-III | 27428 (22138- 31603) | 34380 (14701-44613) | 28880 (23115- 34265) | 0.882 | - |
| *K-Wallis test was performed to show the difference between Placebo, Group A and Group B. Also, the sub-group by dunn test | | | | | | |
